# Anastomotic Leak after Esophagectomy for Esophageal Cancer Treated with a Stent: A Case Report

**DOI:** 10.7759/cureus.4055

**Published:** 2019-02-12

**Authors:** Mohamed Ahmed, Saba Habis, Ahmed Mahmoud, Michael Chin, Rasha Saeed

**Affiliations:** 1 Surgery, Riverside Community Hospital, Riverside, USA; 2 Internal Medicine, Riverside Community Hospital, Riverside, USA

**Keywords:** esophageal cancer, leak, stent, anastamosis

## Abstract

Intrathoracic esophageal anastomotic leaks after cancer resection are very morbid and challenging problems. Esophageal stents play an integral role in the management of these patients. Herein, we present a case of lower esophageal cancer who developed a leak at his gastroesophageal anastomosis after resection and was successfully managed with a fully covered metal stent. Our objective was to remind our colleagues regarding a safe alternative treatment for this complication.

## Introduction

Upper gastrointestinal tract surgery can result in 4% to 8 % anastomotic leaks with a 35% mortality rate [1-3]. Return to the operating room is difficult due to an infected field in morbidly ill patients and carries a risk of further complications. Minimally invasive endoscopic implantation of self-expandable covered stents in the area of the fistula accompanied by drainage can result in healing with minimal morbidity [4].

## Case presentation

**Figure 1:**
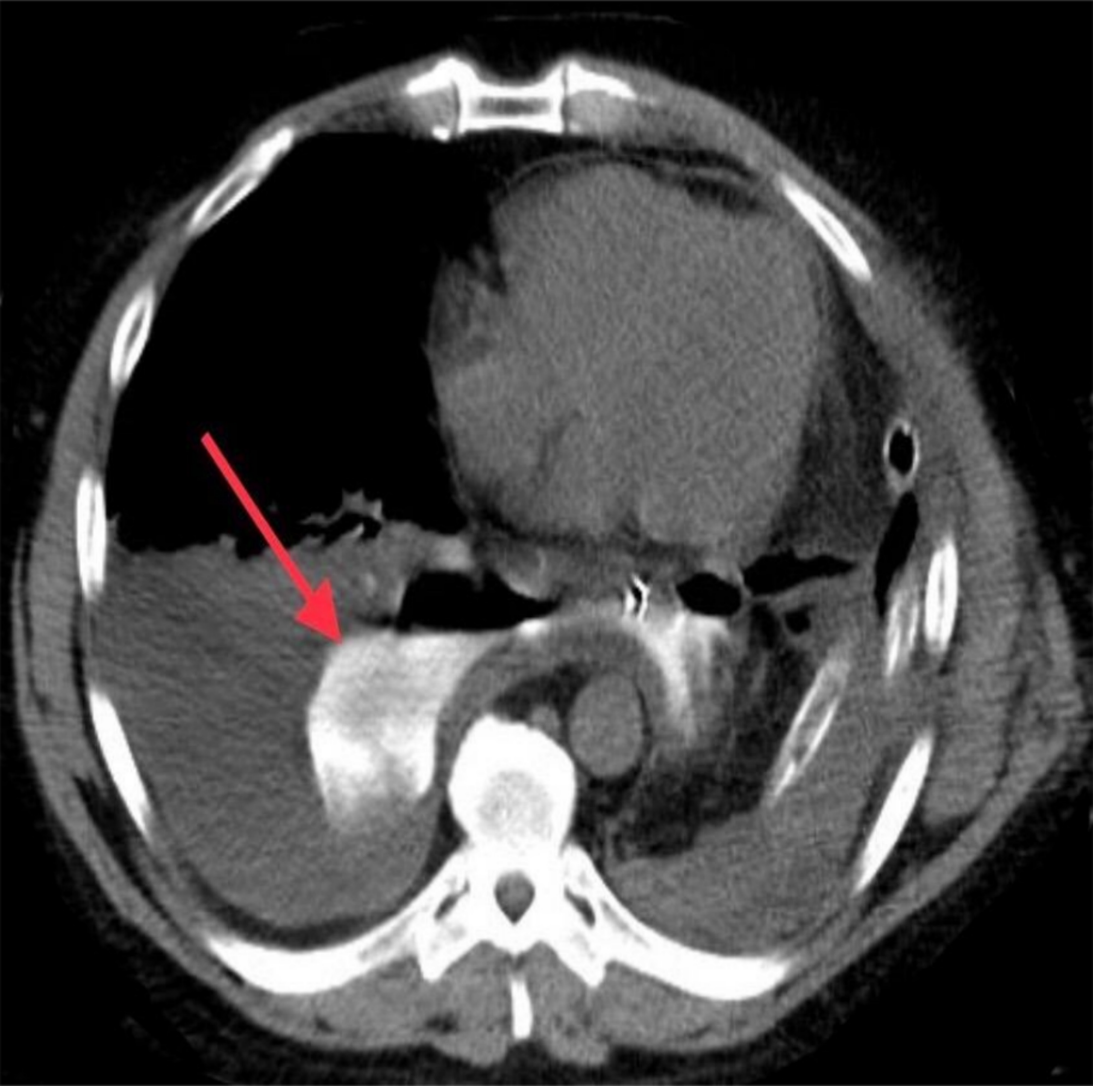
Chest CT scan with oral contrast Anastomotic leak into the right pleural cavity (red arrow)

**Figure 2:**
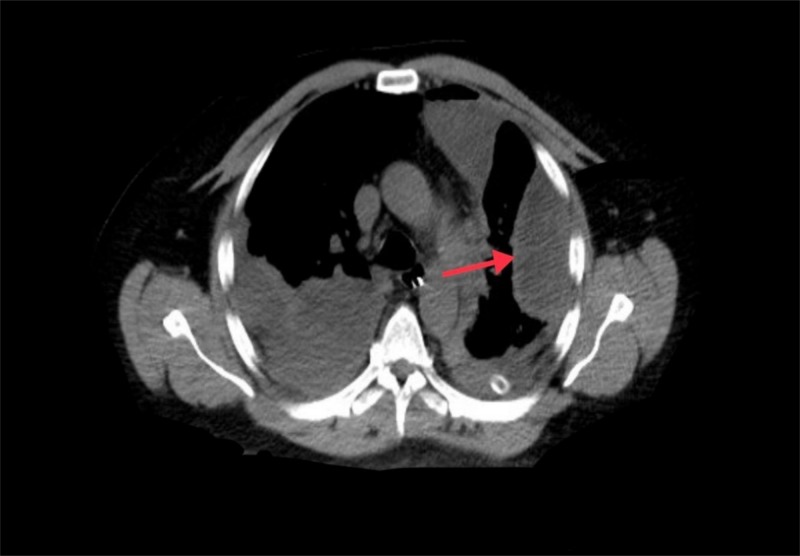
Chest CT scan Loculated abscess (red arrow)

A 58-year-old male underwent Ivor Lewis procedure for a T2N0 well-differentiated adenocarcinoma of the distal esophagus. The patient did well until postoperative day six when he became febrile (temperature of 102 °F) and his white cell count rose to 21 k/mm^3^. Chest computed tomography (CT) scan revealed a frank anastomotic leak (Figure 1) with a loculated abscess formation (Figure 2). 

**Figure 3:**
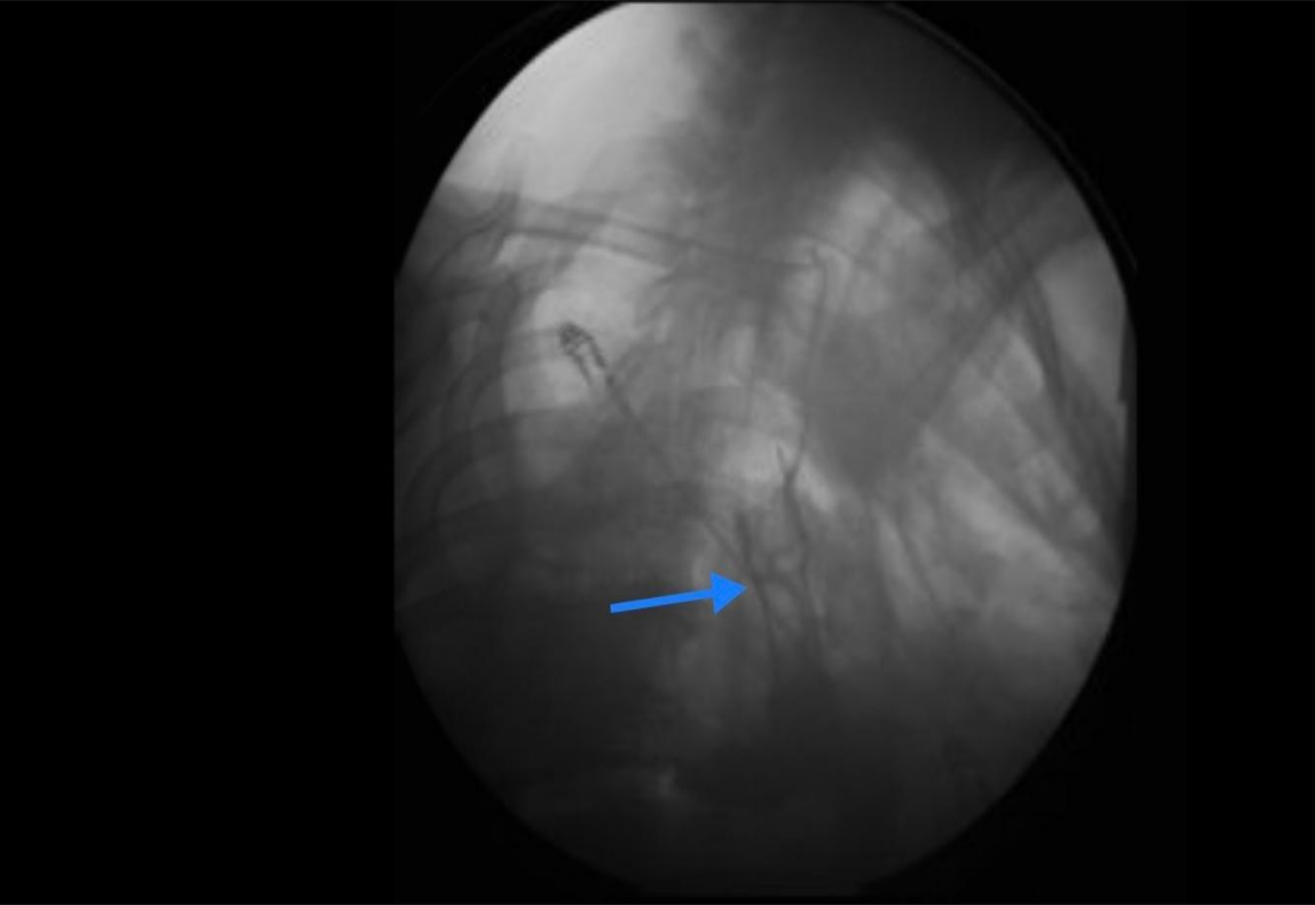
Flouroscopy to guide stent placement Metal stent (blue arrow)

A chest tube was inserted to drain the abscess, control the leak, and obtain microbiological cultures. Broad-spectrum antibiotics coverage was initiated in the form of metronidazole 500 mg intravenous every eight hours and ceftriaxone 2 g intravenous every 24 hours. Esophagogastroscopy revealed a 1-cm disruption at the gastroesophageal anastomosis and a 23-French (diameter), 8-cm (length) fully covered metal stent was deployed by our gastroenterologist to cover the leaking area (Figure 3). 

**Figure 4:**
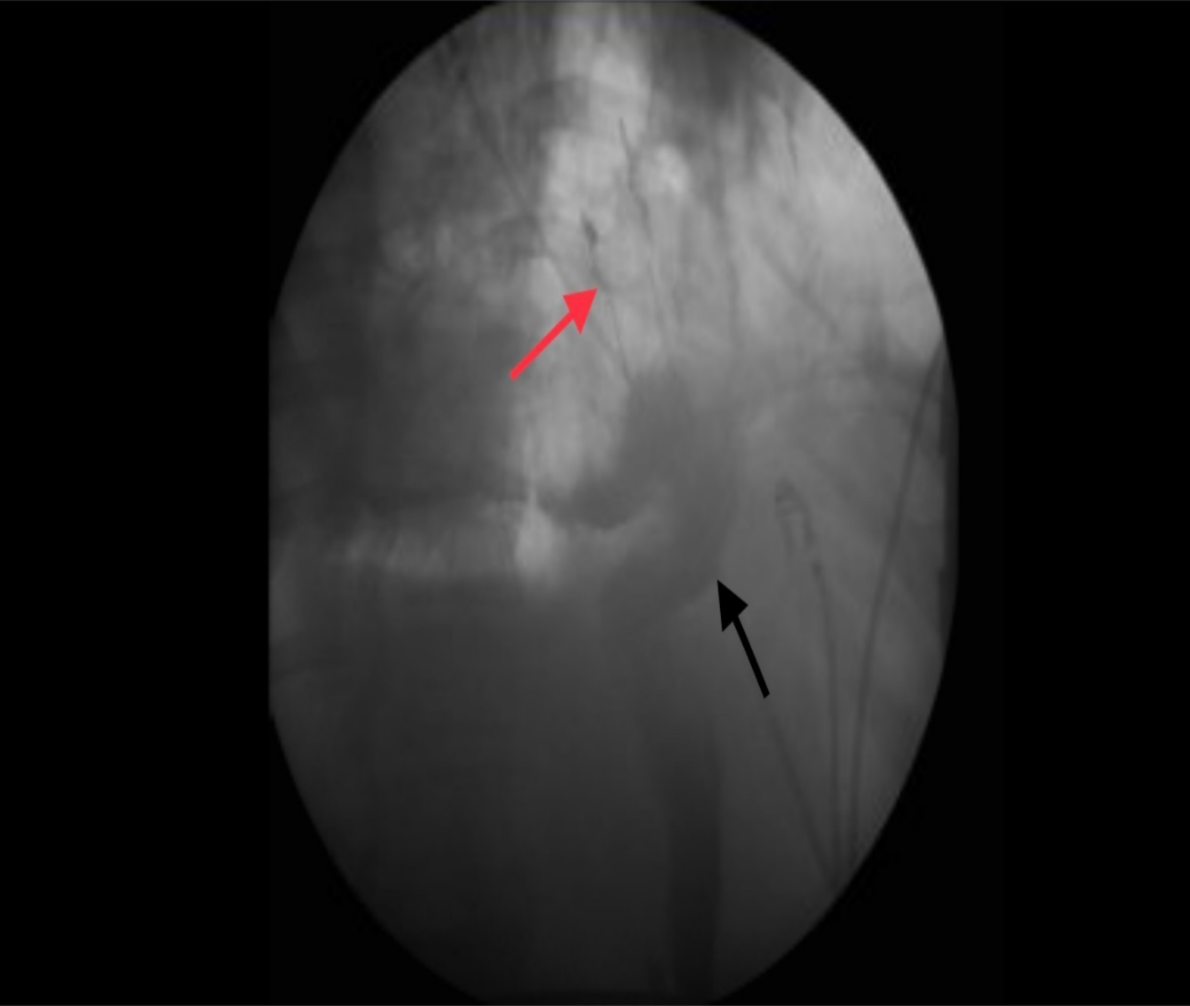
Esophagogram Stent in place (red arrow); stomach remnant (black arrow)

Patient condition dramatically improved after stent placement with normalization of his temperature and white count. Esophagogram a week later demonstrated control of the leak (Figure 4).

The patient did well and was discharged from the hospital. 

## Discussion

Esophagogastric anastomotic leakage is deﬁned as dehiscence of the esophagogastrostomic anastomosis [5]. Risk factors can be subdivided into systemic disease (malnutrition, smoking, diabetes, cardiovascular disease, age, hypoxemia, preoperative chemoradiation, and reduced physical working capacity), operative and postoperative factors (tension at the anastomosis, blood supply, excessive intraoperative bleed, surgeon experience, postoperative distention, and prolonged mechanical ventilation) [6]. The extent of the leak is categorized as contained (small area of contrast extravasation that is contained by mediastinal structures) or uncontained (large leak with contrast freely ﬂowing into the pleural space) based upon appearance on imaging studies [7]. In appropriately selected patients, anastomotic leaks can be managed with non‐operative treatment, whereas extensive leaks from the gastric conduit require revisional surgery that carries a high mortality rate [8].

## Conclusions

Esophageal stents can play an integral role in the management of anastomotic leaks after esophageal resection for cancer. Success depends on adequate drainage of the abscess, appropriate procedures for source control, and in our case, stent placement.
